# The immuno‐reactivity of polypseudorotaxane functionalized magnetic CDMNP‐PEG‐CD nanoparticles

**DOI:** 10.1111/jcmm.16109

**Published:** 2020-11-19

**Authors:** Haiqiang Lan, Tao Huang, Jiangwei Xiao, Zhaohong Liao, Jun Ouyang, Jianghui Dong, Cory J. Xian, Jijie Hu, Liping Wang, Yu Ke, Hua Liao

**Affiliations:** ^1^ Guangdong Provincial Key Laboratory of Construction and Detection in Tissue Engineering Guangdong Provincial Key Laboratory of Medical Biomechanics Department of Anatomy School of Basic Medical Sciences Southern Medical University Guangzhou China; ^2^ UniSA Clinical & Health Sciences University of South Australia Adelaide SA Australia; ^3^ Department of Orthopaedics and Traumatology Nanfang Hospital Southern Medical University Guangzhou China; ^4^ Key Laboratory of Biomaterials of Guangdong Higher Education Institutes Department of Biomedical Engineering College of Life Science and Technology Jinan University Guangzhou China

**Keywords:** CDMNP‐PEG‐CD, immune cells, immune response, Magnetic nanoparticles, β‐CD

## Abstract

pH‐magnetic dual‐responsive nanocomposites have been widely used in drug delivery and gene therapy. Recently, a polypseudorotaxane functionalized magnetic nanoparticle (MNP) was developed by synthesizing the magnetic nanoparticles with cyclodextrin (CD) molecules (CDMNP) via polyethylene glycol (PEG) (CDMNP‐PEG‐CD). The purpose of this study was to explore the antigenicity and immunogenicity of the nanoparticles in vivo prior to their further application explorations. Here, nanoparticles were assessed in vivo for retention, bio‐distribution and immuno‐reactivity. The results showed that, once administered intravenously, CDMNP‐PEG‐CD induced a temporary blood monocyte response and was cleared effectively from the body through the urine system in mice. The introduction of β‐CD and PEG/β‐CD polypseudorotaxane on SiO_2_ magnetic nanoparticles (SOMNP) limited particle intramuscular dispersion after being injected into mouse gastrocnemius muscle (GN), which led to the prolonged local inflammation and muscle toxicity by CDMNP and CDMNP‐PEG‐CD. In addition, T cells were found to be more susceptible for β‐CD–modified CDMNP; however, polypseudorotaxane modification partially attenuated β‐CD–induced T cell response in the implanted muscle. Our results suggested that CDMNP‐PEG‐CD nanoparticles or the decomposition components have potential to prime antigen‐presenting cells and to break the muscle autoimmune tolerance.

## 1 INTRODUCTION

pH‐magnetic dual‐responsive drug delivery is achieved via a particular manner, which could be controlled *via* magnetism and pH responding.^1^, ^2^ Superparamagnetic iron oxide^3^, ^4^, ^5^ is prominently used for cancer therapy and gene therapy.^6^, ^7^, ^8^ Furthermore, these kinds of nanoparticles have been used to control the differentiation of stem cells or monitor biomarkers directly.^9^, ^10^ However, because of precipitation and agglomeration caused by reactive surface, they are not stable.^11^, ^12^ In order to enhance the biocompatibility and stability of the nanoparticles, previous work has utilized various approaches including the use of polyvinyl alcohol,^13^ bovine serum albumin,^14^ polyamidoamine dendrimer^15^ and polyelectrolyte gels.^16^ However, the loading capacity is still low with these modifications, especially for hydrophobic drugs.

Recently, in order to improve the loading capacity of hydrophobic drugs, one kind of nanoparticles was developed, in which an intermolecular aggregation (polypseudorotaxanes) layer was added on magnetic Fe_3_O_4_ core and combined with cyclodextrin (CD) molecules *via* polyethylene glycol (PEG) (CDMNP‐PEG‐CD).^17^ These CDMNP‐PEG‐CD nanocomposites could be used for antibacterial therapy, because they have both magnetic and pH dual‐responsive systems. In vitro tests demonstrated that the nanocomposite was superparamagnetic, cytocompatible, stable in storage and magnetic‐responsive. CDMNP included less roxithromycin (ROX) than CDMNP‐PEG‐CD, and ROX can be released rapidly in the acidic mediums.^17^

Although the side effects of delivery systems in vivo need to be reduced as much as possible, the undesirable toxicity of the synthesized composites to the patients may be triggered in some situations.^18^ For developing the drug delivery carrier, Fe_3_O_4_ core was encircled with β‐CD, which treated by silane coupling agents and further shelled by PEG chains.^17^ As a cyclic oligosaccharide, β‐CD has a character of low pH accelerated degradation.^19^, ^20^ For CDMNP‐PEG‐CD nanocomposites, the antigenicity and immunogenicity of chemically modified β‐CD had been implied in vitro and in vivo.^21^, ^22^ In addition, PEG also was found to help to activate macrophages and lymphocytes in spleen and peripheral blood.^23^, ^24^

The aim of this study was to address the bio‐reactivity of CDMNP‐PEG‐CD nanoparticles in vivo prior to their future explorations of biocompatibility and potential applications. In this study, retention times in different organs and the inflammation/immune responses were assessed for Fe_3_O_4_, Fe_3_O_4_@SiO_2_, Fe_3_O_4_@SiO_2_‐CD and CDMNP@PEG‐CD nanoparticles after their intravenous or intramuscular administration in mice.

## 2 MATERIALS AND METHODS

### 2.1 Ethical approval

All animal experiments were approved by the Animal Experimentation Ethics Committee of Southern Medical University (approval No. L2016068).

### 2.2 Synthesis of magnetic nanoparticles

**Figure 1:**
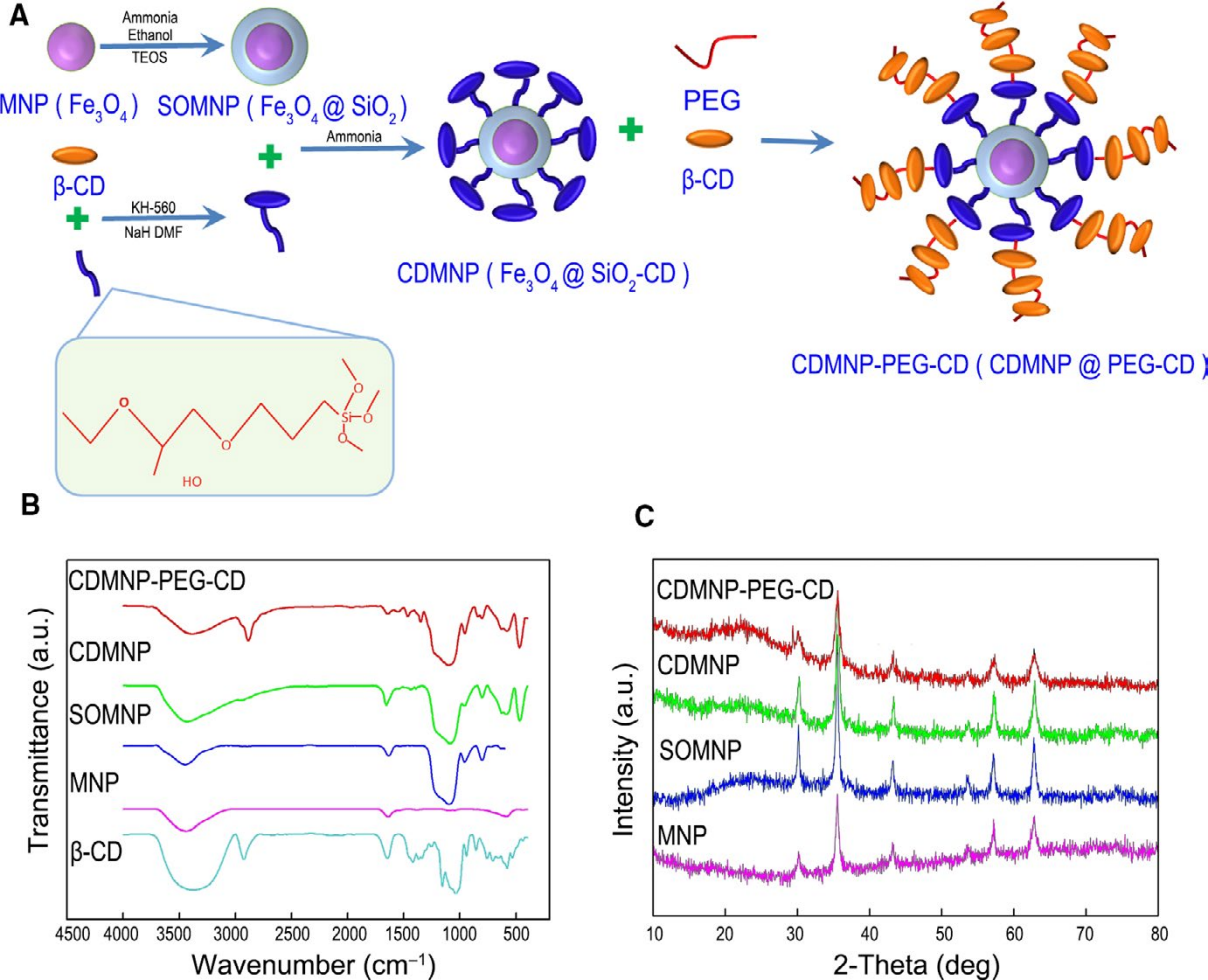
Synthesis of the nanoparticles. A, Synthesis route and structural diagram of CDMNP‐PEG‐CD. B, FTIR spectra of magnetic nanoparticles of MNP, SOMNP, CDMNP and CDMNP‐PEG‐CD. C, XRD patterns of MNP, SOMNP, CDMNP and CDMNP‐PEG‐CD. Reproduced with permission.^17^ Copyright 2019, Elsevier

The structural diagram and synthesis route of Fe_3_O_4_ (MNP), Fe_3_O_4_@SiO_2_ (SOMNP), Fe_3_O_4_@SiO_2_‐CD (CDMNP) and CDMNP@PEG‐CD (CDMNP‐PEG‐CD) are shown in Figure 1A. MNPs were synthesized via a chemical co‐precipitation, where ferrous sulphate and ferric chloride reacted under alkaline condition. Tetraethyl orthosilicate hydrolysed to introduce silicon dioxide layer to produce SOMNP by a sol‐gel approach. β‐CD bonded chemically with epoxy groups of a silane coupling agent (KH‐560), whose methoxy groups at the other side hydrolysed into hydroxy groups and bound with SOMNP to produce siloxane between the organic β‐CD and inorganic SOMNP. The cavity of β‐CD was threaded by a PEG chain; then, more CD molecules were bunched to form a stable polypseudorotaxane for coating on CDMNP *via* self‐association of PEG and CD.

Through chemical co‐precipitation method, bare Fe_3_O_4_ could be obtained ^44^. Briefly, 1.39 g of FeSO_4_.7H_2_O and 2.70 g of FeCl_3_.6H_2_O were mixed in 50 mL water with vigorous stirring at 60°C and pH = 10. After 30 minutes, the mixture was centrifuged and washed. The obtained Fe_3_O_4_ was treated with PEG‐8000 at 80°C. The PEG‐decorated Fe_3_O_4_ could be collected through a magnet. 100 mL 0.01% PEG‐decorated Fe_3_O_4_ dissolved with 80% ethanol. Then, 1 mL of 25% ammonium hydroxide and 0.1 mL of tetraethyl orthosilicate (TEOS) were added dropwise with stirred overnight. The nanoparticles were collected by external magnet. After washed with ethanol and deionized water, the SOMNP nanoparticles were vacuum dried at 60°C overnight.

0.25% NaH and 1.25% β‐CD dissolved in *N*, *N*‑dimethylformamide (DMF) after adding 4 mL KH‐560, and the mixture was heated to 90°C for 5 hours under the protection of nitrogen. Then, 1.5 mL of 25% ammonium hydroxide and 1 g of SOMNP nanoparticles were added slowly. Following stirring at 800 rpm for 12 hours, the brown CDMNP nanoparticles were collected, washed and dried at 50°C.

In order to prepare CDMNP@PEG nanoparticles, CDMNP nanoparticles and PEG (20 vol%) were mixed and stirred at 22°C overnight. CDMNP@PEG was put into DMF with 10‐fold β‐CD. After ultrasonic treatment at 22°C for 10 minutes, the wet pastes were washed and centrifuged at 3200 rpm at the same time.

### 2.3 Nanoparticles characterization

The nanoparticles were tested through Fourier transform infrared spectrometry (FTIR, Bruker, Germany) and X‐ray diffractometer (Netherlands). All nanoparticles were treated as required. The detection was performed from 10° to 80° of 2θ with 5°/min ration through Malvern Zetasizer Nano ZS (Britain). Through dynamic light scattering equipment and electrophoretic light scattering, hydrodynamic diameters and surface charge of the nanoparticles were obtained. Hitachi S‐4800 field emission scanning electron microscope (SEM, Japan) and FEI Tecnai G2 F20 microscope were used to observe the surface and transmission electron microscopy (TEM) picture of CDMNP‐PEG‐CD. TEM samples were prepared as required. Thermogravimetry and derivative thermogravimetry (DTG) curves were performed by Netzsch (Germany) 209F3‐ASC thermogravimetric analyzer.

In order to investigate the magnetic properties of the nanocomposites, magnetometer (VSM, USA) was used to evaluate and record hysteresis loops from −20 to 20 kOe. We also tested the magnetic response by external magnet. 0.02 g of nanocomposites was added into 12 mL water; after treating with ultrasonic for 0.5 hours, the stability was detected.

For assessing fluorescence loading of the particles, FITC was bound on the nanoparticles *via* the catalysis of sodium hydride. The FITC‐bound nanoparticles were washed by deionized water and detached by a magnet, till no UV absorption of washing liquid was detected. The bound amounts of FITC were calculated based on the UV absorption of the FITC solution and according to the calibration curves (*R* ^2^ = 0.999).

### 2.4 In vivo bio‐distribution

C57BL/6 mice of 8 weeks were used in in vivo experiments. The animals were distributed in 3 experimental groups (n = 6), corresponding to SOMNP, CDMNP and CDMNP‐PEG‐CD. The mice were anaesthetized (isoflurane 2%) and then injected via the tail vein with nanoparticle diluted in phosphate‐buffered solution (PBS) 200 μL, which were pre‐bound with green FITC fluorescent dye. The mice of control group were treated with PBS. On 12 and 24 hours after intravenous injection, cardiac puncture was performed for blood collection. Then, the mice were killed by CO_2_ overdose after the blood collection. Liver, spleen, kidney and mesenteric lymph nodes were removed. The non‐invasive organ fluorescence imaging analysis FX Pro (Bruker, Billerica, USA) was used to monitor distribution and retention of the FITC labelled nanoparticles in different organs.

Mouse blood was collected and incubated with RBC Lysis Buffer (eBioscience, San Diego, CA, USA) follow its instruction. The samples were centrifuged at 500 *g* for 5 minutes at 22°C. The cells were collected in Flow Cytometry Staining Buffer (eBioscience, San Diego, CA).

### 2.5 Muscle injection of the nanoparticles and sample collection

Under anaesthetized with ketamine, animals were injected with 50 μL PBS suspension containing SOMNP, CDMNP or CDMNP‐PEG‐CD 300 μg, respectively, into the gastrocnemius muscle (GN). Mice received only PBS injecting were taken as the control. On days 1, 3, 5, 7 and 10 post‐injection, GN muscle samples, popliteal and inguinal draining LNs were collected. Muscle samples were detected by histological staining. Muscle samples and LNs were minced and digested by II collagenase for fluorescence‐activated cell sorting (FACS).

### 2.6 Histological and immunofluorescence staining

GN muscle containing nanoparticles was cryosectioned at 8 mm transversely, followed by haematoxylin and eosin (H&E) staining or immunofluorescence staining. Muscle sections were incubated with rat antimouse F4/80 (1:200; eBioscience) after fixed and followed by incubation with Cy3‐labelled goat anti‐rat IgG (1:500; Beyotime). Olympus BX51 fluorescence microscope (Olympus) were used for viewing after treated with DAPI (Abcam).

### 2.7 Flow cytometric analysis

Spleen and LNs of mice received the muscle nanoparticles implants were collected. Muscle was dissociated in DMEM with collagenase II (0.2%, Sigma‐Aldrich) at incubator for 45 minutes. After washing and blocking, cells were incubated with antibodies. A FACSCalibur flow cytometer (BD, Franklin Lakes, USA) and FlowJo software (Tree Star, Inc) were used for detection. The following Abs were used: PACIFIC BLUE‐conjugated Abs against CD45 (1:100; eBioscience), APC‐conjugated Abs against CD3 (1:100; eBioscience), APC‐CY7‐conjugated Abs against CD4 (1:100, eBioscience), PE‐conjugated Abs against F4/80 (1:100, eBioscience), PE‐conjugated Abs against CD8a (1:100, eBioscience) and PE‐conjugated Abs against CD11b (1:100, eBioscience).

### 2.8 Adoptive transfer and analysis of T cell priming

CD45.1^+^ TCR‐Tg OT‐I mice provided the OVA‐specific CD8^+^ T cells, which were purified by negative magnetic sorting (Miltenyi Biotec, Bergisch Gladbach, Germany), and labelled with intracellular CFSE dye (Invitrogen). The phenotype of the purified cells was assessed by flow cytometry (Beckman Coulter, Brea, USA). Transgenic MCK3E‐OVA mice, expressing a membrane‐bound form of ovalbumin (OVA) exclusively in skeletal muscle, received nanoparticle implanting by im injection 50 μL and followed by tail vein injection 200 μL of CFSE‐labelled OT‐I cells next day. Mice that underwent T cell but not nanoparticle implanting were control mice. For positive control, T cell transfer was performed 1 day before soluble OVA (10 μg per mouse; Sigma‐Aldrich) injected into GN muscle of the mice. On day 4 post‐implant, CD45.1^+^CD8^+^ cells were collected from muscle draining LNs (dLNs) of the recipient mice, and the proliferation was measured by FACS.

### 2.9 Statistical analysis

SPSS ver.13.0 software (IBM, Armonk, USA) was used for statistical analysis. To multiple comparisons, one‐way analysis of variance (ANOVA) was used. All results were recorded as mean ± standard deviation (SD). When *P* values < .05, the data were considered significant.

## 3 RESULTS

### 3.1 Structure and characterization of the nanoparticles

Figure 1A demonstrated synthesis route and structural diagram of the nanoparticles. MNP presented two strong peaks at 590 cm^−1^ and 1628 cm^−1^, respectively, corresponding to the absorption of Fe‐O tetrahedron and the bending vibration of ‐OH groups. SOMNP showed two additional peaks at 797 cm^−1^ and 1096 cm^−1^ of the symmetric and asymmetric stretching vibration of Si‐O‐Si. Because of the bending vibration of ‐OH and ‐CH of β‐CD, CDMNP had two small peaks around 1435 cm^−1^ and 1387 cm^−1^ compared with SOMNP. A strong peak at 2871 cm^−1^ of the terminal ‐OH of PEG and two peaks at 1461 cm^−1^ and 1357 cm^−1^ of the bending vibration of β‐CD in CDMNP‐PEG‐CD demonstrated that PEG and β‐CD were introduced in the layer on magnetic core (Figure 1B). The structure of synthesized particles was evaluated by XRD patterns, which showed that the crystal structure of magnetite nanoparticles core did not change after the formation of the shell (Figure 1C).

**Figure 2:**
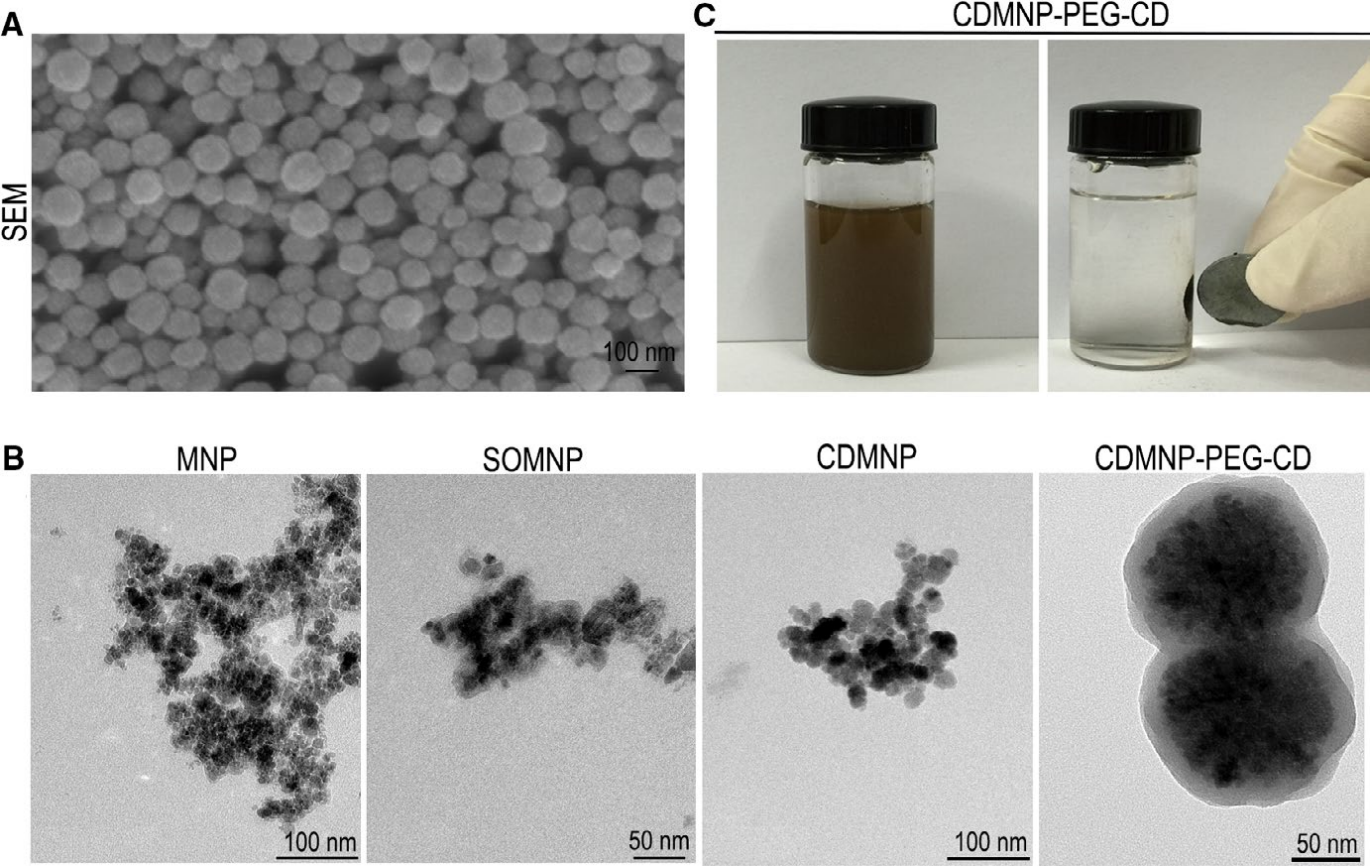
The shape and characterization of the nanoparticles. A, SEM image of CDMNP‐PEG‐CD. B, TEM images of MNP, SOMNP, CDMNP and CDMNP‐PEG‐CD. C, The magnetics of CDMNP‐PEG‐CD

Being coated with hydrophilic layers, CDMNP‐PEG‐CD nanoparticles were distributed in spherical shape. The nanoparticles presented obvious core (magnetite clusters)‐shell (SiO_2_, PEG or CD) structure, and their size increased orderly (Figure 2A,B). Fe_3_O_4_ nanoparticles were prone to agglomerate and precipitate in aqueous solution, but CDMNP‐PEG‐CD colloid after 30 minutes ultrasonic agitation was stable even at 8‐minute storage (Figure 2C), showing the enhanced dispersive capacity of CDMNP‐PEG‐CD via polypseudorotaxanes layer. It responded to an external magnet and aggregated on the wall within 30 seconds (Figure 2C). The results of the magnetic responsiveness and thermal gravimetric analysis of the dispersed nanocomposites were consistent with our previous report.^17^

### 3.2 In vivo bio‐distribution and retention assessment of the nanocomposites

**Figure 3:**
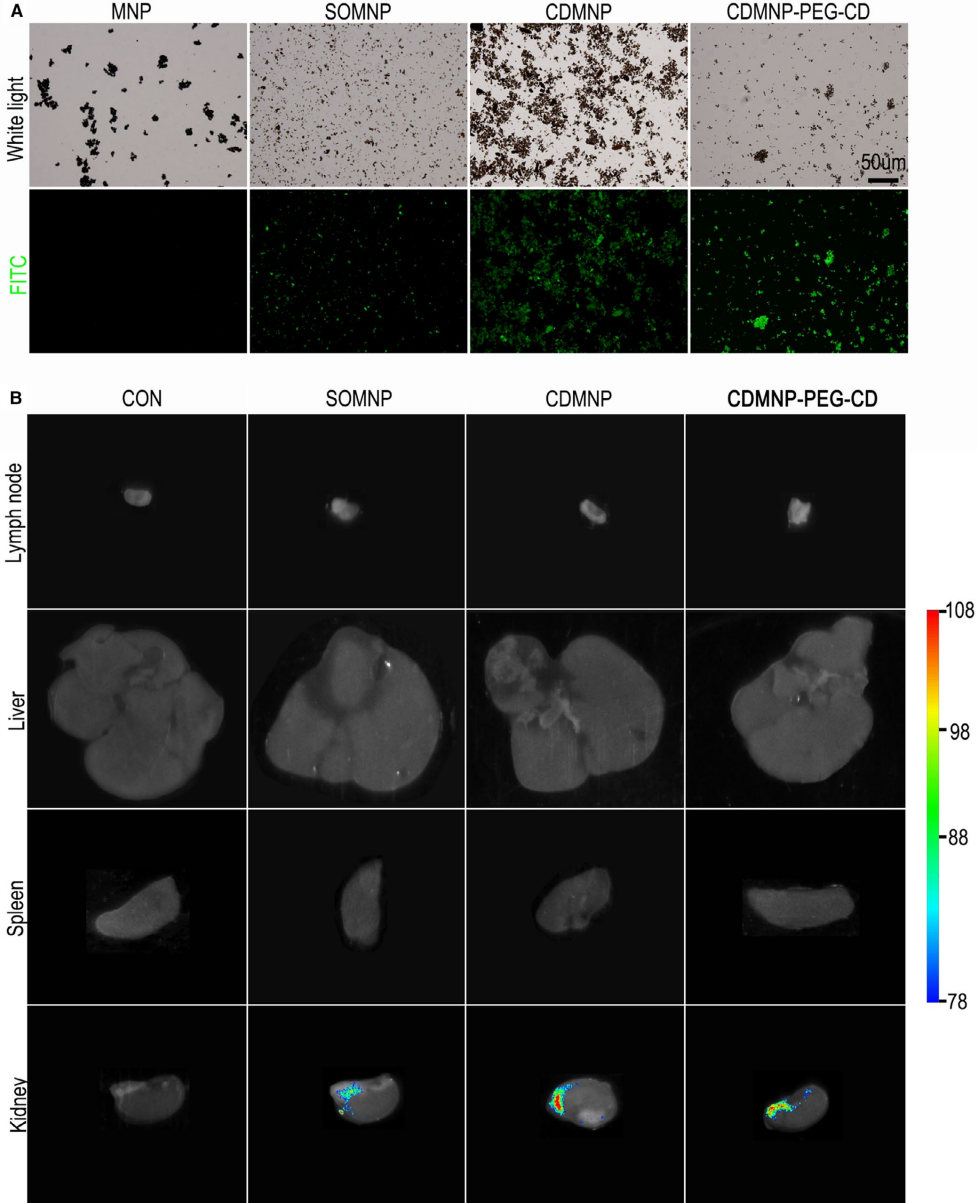
In vivo bio‐distribution and retention of the nanoparticles. FITC binding of MNP, SOMNP, CDMNP and CDMNP‐PEG‐CD. B, Distribution of the nanoparticles in mouse lymph nodes, liver, spleen and kidney as monitored by organ fluorescence imaging analysis. Bar = 50 μm

Considering that intravenous route is the most commonly used way of drug administration in vivo, B6 mice were firstly implanted with the nanoparticles by tail vein injection, which were pre‐bound with green FITC fluorescent dye (Figure 3A). The mass ratio of FITC to the nanoparticles was 0.122%, 0.136% and 0.232% for SOMNP, CDMNP and CDMNP‐PEG‐CD, respectively. These ratios were then multiplied by the mass of different nanoparticles in PBS, resulted in the administration amount of FITC for SOMNP, CDMNP and CDMNP‐PEG‐CD. The non‐invasive organ fluorescence imaging analysis was used to monitor distribution and retention of the nanoparticles in different organs. Noteworthy, 12 hours after intravenous injection of the FITC‐binding nanoparticles, nanoparticles trapped were not found in liver, spleen and mesenteric lymph nodes of the exposed mice (Figure 3B). Exceptionally, a little number of green particles could be observed in renal pelvis area of kidneys (Figure 3B), suggesting the nanoparticles rapidly and effectively excreted out of the body through mice urinary system, in the absence of the external magnetic field. Meanwhile, no significant differences of the three particles retention in liver, spleen and lymph nodes, SOMNP cleared from kidney more rapidly than CDMNP and CDMNP‐PEG‐CD on 12 hours post‐injection (Figure 3B). This indicates that the adding of β‐CD or PEG, or their intermolecular aggregation upon Fe_3_O_4_ magnetic particles may affect on renal filtration for the prepared nanocomposites.

**Figure 4:**
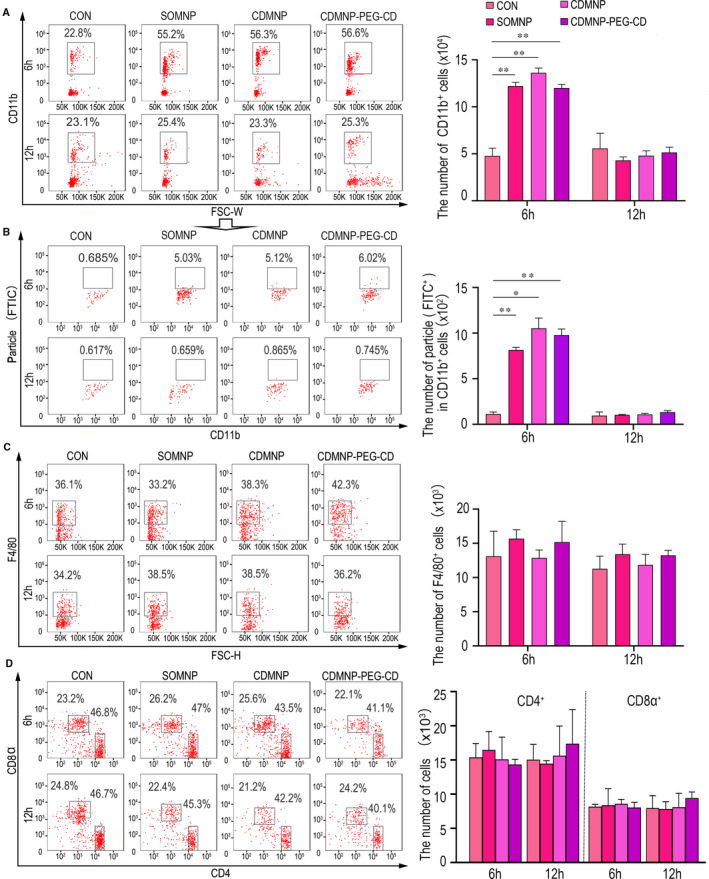
FACS analysis of immune cell response to the nanoparticles in vivo. In blood, CD11b^+^ monocytes (A) and their phagocytosis efficiency (B) were detected. F4/80^+^ macrophages (C), CD4^+^ and CD8^+^ T cells (D) in spleen were presented after intravenous injecting of nanoparticles. All data are presented as mean ± SD (n = 3). One‐way ANOVA was used for multiple comparisons. (*P < .05, **P < .01)

To further understand whether the MNPs provoke monocyte response, the changes in number and function of blood monocytes after intravenous injecting of nanoparticles were next investigated *via* FACS analysis. The results showed a higher proportion of CD11b^+^ cells, which presented the elevated phagocytosis efficiency, in peripheral blood of animals 6 hours after particle injection, comparing to that of untreated animals (Figure 4A,B, Figure S1A,B). However, blood monocytes number and function returned to the normal 12 hours after injecting. Consistent with the result of particle retention in organs, no statistical significance of monocyte number and phagocytosis function was monitored among animals received SOMNP, CDMNP or CDMNP‐PEG‐CD, respectively (Figure 4A,B). In spleen, no markedly difference of the absolute number of macrophages (F4/80^+^) and T cells(CD3ε^+^CD4^+^; CD3ε^+^CD8α^+^) were found among the untreated and nanoparticles exposed mice (Figure 4C,D, Figure S1C). The data indicate that the developed MNPs are basically immune and biological compatible in vivo, which can be cleared from the body in time, and only trigger a weak and transient systematic immune reaction.

### 3.3 The local inflammation response induced by the nanocomposites

**Figure 5:**
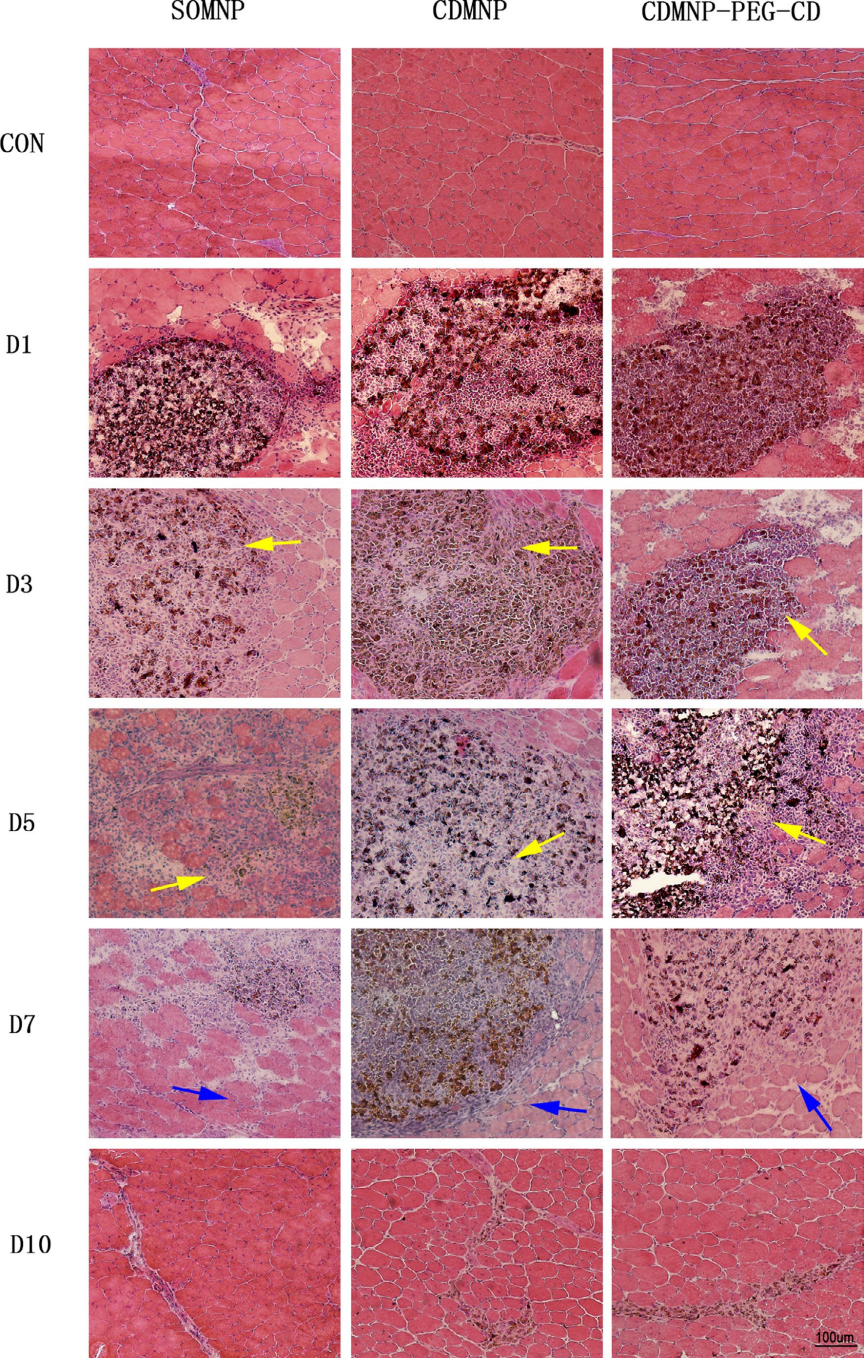
The nanoparticles induce intramuscular inflammation response. The representative images of H&amp;E staining of gastrocnemius muscle containing the nanoparticles, revealing that, while SOMNP has widely dispersed on day (D) 3 post‐injection, the particle dispersion was observed on day 3 for CDMNP and day 5 for CDMNP‐PEG‐CD. Areas of inflammation indicated by yellow arrows, and areas of regeneration indicated by blue arrows. Bar = 100 μm

The muscle toxicity and local immunogenicity of the nanocomposites injected into mice GN muscle were explored for considering the importance of intramuscular injection in drug treatment. HE staining of GN muscle containing the implanted particles was checked. As shown in Figure 5, 1 day after injecting im, the brownish nanoparticles can be observed to aggregate in GN muscle, which induced a conspicuous mononuclear cell infiltration surrounding the nanoparticles. Over time, implanted nanoparticles gradually dispersed and completely disappeared on day 10 post‐injecting.

Interestingly, SOMNP dispersed more quickly and induced a more severe inflammation infiltration during the early implanting phage (days 1 and 3) than that of CDMNP and CDMNP‐PEG‐CD. SOMNP was cleared effectively and muscle inflammation resolved on day 7 post‐injecting. On the contrary, in muscle tissue, the obvious particle dispersion was observed from day 3 for CDMNP, and from day 5 for CDMNP‐PEG‐CD, implying that the modification via β‐CD and PEG/β‐CD polypseudorotaxane increased tissue retention of the nanocomposites. Inflammatory infiltration and muscle fibre degeneration appeared in CDMNP‐ and CDMNP‐PEG‐CD–injected muscle on days 3, 5 and 7 post‐injecting, following the nanoparticle dispersing.

**Figure 6:**
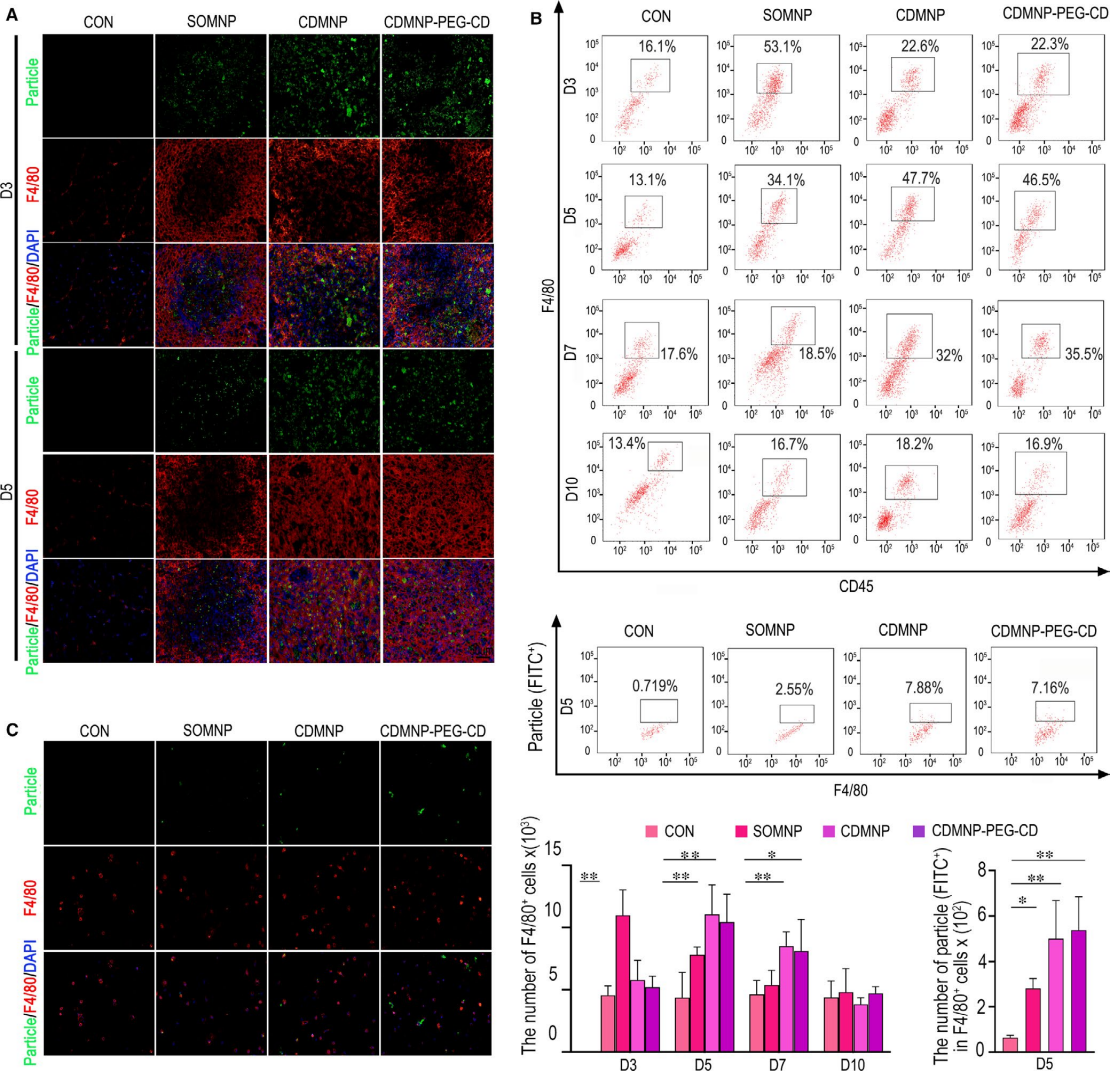
The nanoparticles induce macrophage response in vivo and in vitro. A, Representative fluorescence staining of F4/80^+^ macrophages in GN muscle on days 3 and 5 post‐injecting of nanoparticles. B, FACS analysis of CD45^+^F4/80^+^ cell population in nanoparticle‐injected muscle (upper part). Phagocytosis efficiency of F4/80^+^ cells on day 5 was monitored (lower part). C, Representative fluorescence staining of F4/80^+^ cells engulfing SOMNP, CDMNP and CDMNP‐PEG‐CD in vitro, which were obtained from mouse abdominal cavity. All data are presented as mean ± SD (n = 3). One‐way ANOVA was used for multiple comparisons. (*P < .05, **P < .01). Bar = 50 μm

Immunostaining and FACS analysis were conducted to analysis CD45^+^F4/80^+^ cells in GN muscles which received nanoparticles. Under fluorescence microscope, a dramatic increase of F4/80^+^ macrophages in GN muscle was observed on days 3 and 5 post‐injecting of nanoparticles, comparing to that of untreated muscle. The peak of F4/80^+^ cell infiltration occurred on day 3 for SOMNP, but on day 5 for CDMNP and CDMNP‐PEG‐CD (Figure 6A). Compared to control muscle, the obvious increase of CD45^+^F4/80^+^ cells was detected in nanoparticle‐injected muscle by FACS (Figure 6B). In agreement with immunostaining results, we found a higher proportion of F4/80^+^ cells in SOMNP‐contained muscle on day 3 post‐injecting, but in CDMNP‐ or CDMNP‐PEG‐CD–contained muscles on day 5. At the day 7, a small number of monocytes/macrophages can be monitored in CDMNP‐ and CDMNP‐PEG‐CD–contained muscle (Figure 6B, Figure S2). In addition, F4/80^+^ cells engulfed more CDMNP‐PEG‐CD, than CDMNP and SOMNP in vivo (Figure 6A,B) and in vitro (Figure 6C).

### 3.4 The nanocomposites trigger T cell reaction in muscle and in muscle draining lymph node

**Figure 7:**
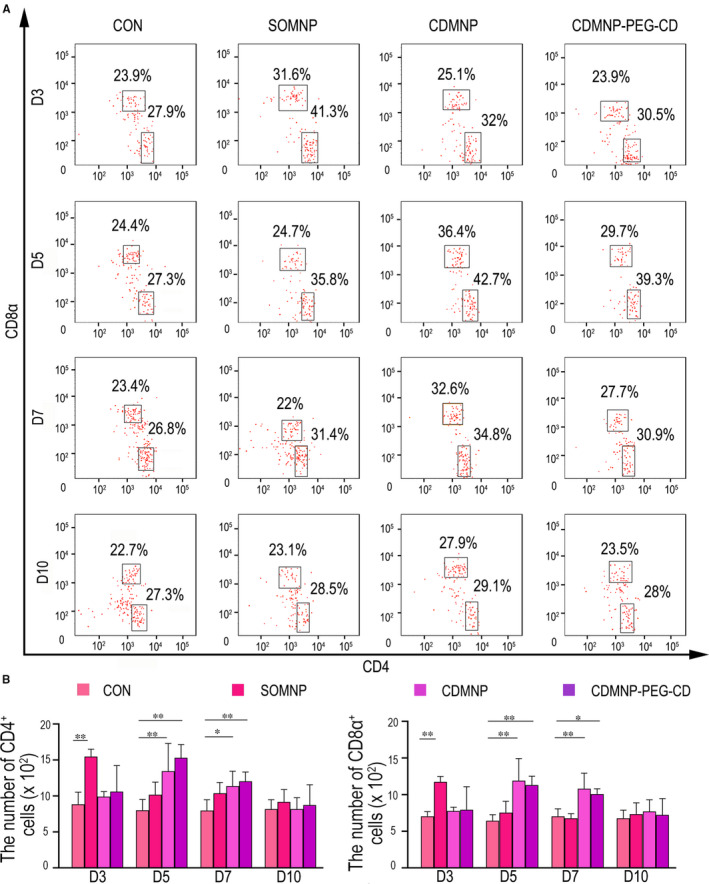
The nanoparticles induce T cell reaction in muscle. A, FACS analysis for the infiltration of CD4^+^ and CD8^+^ T cells in GN muscle embedded with SOMNP, CDMNP and CDMNP‐PEG‐CD particles (CD3^+^ cells were firstly gated from particle‐treated muscle). B, Statistical analysis of CD4^+^ and CD8^+^ T cells. All data are presented as mean ± SD (n = 3). One‐way ANOVA was used for multiple comparisons. (*P < .05, **P < .01)

To further address antigenicity of the implanted nanocomposites, FACS analysis was performed to detect T cell infiltration in the nanocomposite‐embedded muscles and in muscle draining lymph nodes (dLNs). As shown in Figure 7A,B, and Figure S3, all three nanoparticles attracted CD3^+^CD4^+^ and CD3^+^CD8^+^ T cells to invade into GN muscle after particle injecting. The highest T cell infiltration was monitored on day 3 for SOMNP, but on day 5 for CDMNP and CDMNP‐PEG‐CD. At the same detecting time‐point, CDMNP induced the more severe T cells infiltration than SOMNP and CDMNP‐PEG‐CD, suggesting T cell is more susceptible for β‐CD–modified magnetic particles than SiO_2_‐Fe_3_O_4_ core, and it is possible that CDMNP‐induced T cell activation can be partly blocked by the PEG chains exposing outside β‐CD cavities.

T cell activation in muscle dLNs on days 3 and 5 post‐nanoparticle implanting were investigated because the nanoparticle may be captured by the infiltrated immune cells and removed to muscle dLNs to activate T cells and prime the adaptive immunity. However, we found that in popliteal and gluteal lymph nodes of nanoparticle administrated mice, there was no significant difference between experimental group and control group on the number of CD4^+^ and CD8^+^ T cells (data not shown).

**Figure 8:**
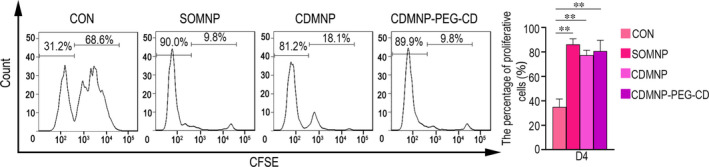
FACS analysis for the proliferation of the purified CFSE‐labelled CD45.1^+^CD8^+^ OT‐I cells in muscle dLNs in MCK3E‐OVA Tg mice that received nanoparticle injection (day 4 post‐injection). All data are presented as mean ± SD (n = 3). One‐way ANOVA was used for multiple comparisons. (*P < .05, **P < .01)

To directly clarify whether nanoparticle triggered‐muscle degeneration and necrosis effects on muscle self‐tolerance, purified CFSE‐labelled OVA‐specific CD8^+^ T cells from TCR‐Tg OT‐I mice harbouring the CD45.1^+^ allelic variant were adoptively transferred, to MCK3E‐OVA Tg mice, which could selectively express ovalbumin (OVA) as a self‐antigen in skeletal muscle. MCK3E‐OVA Tg mice received OT‐I cell transfer one day after nanoparticle injection im or not. OT‐I cell proliferation in muscle dLNs on day 4 post‐particle injection was detected. All three nanoparticles led to OT‐I cell expansion in muscle dLNs of MCK3E‐OVA recipient mice (Figure 8). However, no significant difference of OT‐I cell priming in muscle dLNs was monitored among SOMNP, CDMNP and CDMNP‐PEG‐CD (Figure 8).

## 4 DISCUSSION

External implants usually cause immune and inflammatory responses in vivo. The inflammation directly affects the compatibility and stability of biomaterials, and thus can affect the functional efficacy of biomedical devices. Recently, much attention has been paid to the nanoparticles, which were taken as the effective drug delivery devices.^25^ Bioaccumulation and immune response of inorganic gold nanoparticles^26^ and carbon nanoparticles^27^ had been studied. However, systematic and local antigenicity and immunogenicity primed by nanoparticles in vivo are yet to be dissected fully. In this study, the immune toxicity and bio‐distribution of CDMNP‐PEG‐CD nanoparticles were investigated in mice after administration by intravenous or intramuscular injection. Under intravenous administration, the nanoparticles can induce a temporary blood monocyte response and can be cleared effectively from the body through the urine system. In the implanted muscle, T cells are more susceptible for β‐CD–modified CDMNP, but polypseudorotaxane modification reserves β‐CD–induced T cell response. Our findings have revealed that CDMNP‐PEG‐CD particles or their decomposition components have potential to prime APCs and to break muscle autoimmune tolerance.

To determine the toxicity and kinetics of nanoparticles, the functional coating of nanoparticles is a key factor. β‐CD chemically functionalized the magnetic iron oxide nanoparticles, and then, large numbers of CD molecules were threaded by PEG chains to get the polypseudorotaxanes coating of CDMNP‐PEG‐CD.^17^ After intravenous injection, the liver,^26^, ^27^, ^28^ spleen or lung trapped the nanomaterials.^29^, ^30^, ^31^ In the kidney, small particles of nanomaterials might also accumulate for renal excretion.^28^ Here, the results of the organ imaging showed efficient and fast particle excretion in urine one day following the injection. For in vivo application, the most important thing is the hydrodynamic size and the circulation time of magnetic particles. When there is a hydrophilic coating on the nanoparticles, the site specificity is enhanced through avoiding from being up‐taken by the reticuloendothelial system.^32^, ^33^ In our previous work, it was about ~45 nm for the size of SOMNP in aqueous solution, at the same time the hydrodynamic diameters of CDMNP and CDMNP‐PEG‐CD was 83 nm and 167 nm. As MNP diameter was about 22nm, the thickness of coating could be calculated as 23 nm for SOMNP, 61 nm for CDMNP and 145 nm for CDMNP‐PEG‐CD. In addition, on the basis of the yields, the mass ratios of Fe_3_O_4_/CD for CDMNP and CDMNP‐PEG‐CD were nearly 2.0 and 0.57, respectively, and that of Fe_3_O_4_/PEG was 1.6 in CDMNP‐PEG‐CD.^17^ The larger size particles were formed by hydrophilic coating, different zeta potential, and of the changed aggregation ability by introduction of SiO_2_, β‐CD and PEG/β‐CD polypseudorotaxane. The current study demonstrated that the removal of CDMNP‐PEG‐CD by the body is not different from that of SOMNP and CDMNP, in the absence of the external magnetic field.

For investigating the immune susceptibility of the developed nanoparticles in vivo, the number and function changes of blood monocytes were monitored after intravenous injection of the nanoparticles. It was confirmed that all three nanoparticles were effectively cleared from the blood circulation during 12 hours following the injection. However, at the early stage after injection (4 hours), the number of blood monocytes increased and phagocytosis ability was elevated. This result implies a weak and transient immune reaction induced by the nanoparticles, which was further supported by the negative number changes of macrophages and T cells in the spleen. Previously, Jong et al found that gold nanoparticles with a diameter between 50 and 200 nm were mainly detected in the blood. When the size was reduced to 10 nm, they were largely detected in the spleen and liver.^34^ In the current study, the larger CDMNP‐PEG‐CD particles can be cleared from the body effectively from blood circulation as the smaller CDMNP and SOMNP particles. In the current study, in order to keep nanoparticles from opsonization, PEG was used, which was found to have enabled the circulation time in blood and organ accumulation of the nanoparticles increased.^35^ Nevertheless, our data showed that the surface functionalization of Fe_3_O_4_ particles by β‐CD and PEG/β‐CD polypseudorotaxane, which had increased the particle hydrodynamic diameter and coating thickness, did not result in the more severe or unusual response of peripheral blood monocytes.

With the aqueous solubility of β‐CD being restricted, the dispersion of CDMNP‐PEG‐CD particles in water was strongly enhanced by the necklace‐like crystalline polypseudorotaxanes layer as physical crosslinking sites.^17^ However, following intramuscular injection, H&E staining analyses showed that the introduction of β‐CD and PEG/β‐CD polypseudorotaxane on SOMNP increased aggregation of the nanoparticles in GN muscle, especially for CDMNP‐PEG‐CD. The rapidly dispersed SOMNPs were engulfed by infiltrated macrophages or removed *via* muscle vascular system. Therefore, SOMNP‐induced muscle inflammation resolved rapidly. On the other hand, delayed intramuscular dispersing of CDMNP and CDMNP‐PEG‐CD accordingly postponed immune cell infiltration and their functional activation, resulting in the prolonged local immune response. These extended inflammation phenomena were further supported by FACS analyses in this study, which found that the accumulation peak of macrophages and T cells in CDMNP or CDMNP‐PEG‐CD embedded muscle appeared on the day 7 post‐injection, about 2 days later than that in SOMNP (on day 5 post‐injection). As a drug delivery carrier, magnetic nanoparticles are required to stay in the target tissues or organs long enough so to release a special drug for disease treatment, by bearing external magnetic field. Therefore, future work is necessary to address how to avoid the sustained immune toxicity triggered by the nanocomposites persistently retained in vivo.

Food and Drug Administration (FDA) has approved the superparamagnetic iron oxide nanoparticles with silicone as bio‐compatible and clinically acceptable product.^36^ Our in vitro tests demonstrated that CDMNP and CDMNP‐PEG‐CD performed much better than SOMNP in cytocompatibility and that the cell viability of CDMNP‐PEG‐CD showed much higher than the other two nanocomposites.^17^ Through in vivo testing, myocyte degeneration and necrosis surrounding the aggregated nanoparticles were observed in all three nanoparticles embedded muscles, centronuclear new myofibres rapidly appeared and replaced the necrotic myofibres following the nanoparticles dispersion and inflammation resolution. This phenomenon suggests that SOMNP, CDMNP and CDMNP‐PEG‐CD are relatively bio‐compatible in vivo.

In some myopathic conditions, muscle antigens can be released from degenerated myofibres, captured by antigen‐presenting cells (APCs, such as macrophages) and transferred to muscle draining lymph nodes (dLNs) for priming autoimmune T cells.^37^, ^38^ In addition, there is growing evidence that nanoparticles can affect adaptive immunity, particularly by affecting the function of APCs.^39^, ^40^, ^41^ Here, T cells were found to accumulate in nanoparticle‐embedded GN muscle, which could reflect that implanted nanoparticles possibly affect muscle self‐tolerance through interfering with the local inflammation environment. We addressed this problem and monitored the priming of OVA‐specific CD8^+^ OT‐I T cells in muscle dLNs at the early stage (day 4) after nanoparticles were injected im Surprisingly, no significant differences were found between SOMNP, CDMNP and CDMNP‐PEG‐CD in OT‐I cell initiation in muscle dLNs. In vivo, nanoparticles could be recognized by immune cells, leading to the release of pro‐inflammation mediators (ie IL‐1, IL‐6).^29^, ^42^, ^43^ It can be speculated that before the dispersion and clearance, MNPs entrapped in muscle or their decomposition elements may induce immune cells to release some special pro‐inflammatory cytokines or chemokines, which result in the break of muscle autoimmune tolerance.

## CONFLICT OF INTEREST

The authors have declared that no conflicts of interest.

## AUTHOR CONTRIBUTIONS

**HaiQiang Lan:** Data curation (lead); formal analysis (lead); writing‐original draft (equal). **Tao Huang:** Data curation (equal); formal analysis (equal); writing‐original draft (equal); writing‐review and editing (equal). **Jiangwei Xiao:** Data curation (equal); investigation (equal). **Zhaohong Liao:** Data curation (equal); investigation (equal). **Jun Ouyang:** Data curation (equal); formal analysis (equal). **Jianghui Dong:** Data curation (equal); formal analysis (equal); writing‐original draft (equal); writing‐review and editing (equal). **Cory Xian:** Writing‐original draft (equal); writing‐review and editing (equal). **Jijie Hu:** Data curation (equal); formal analysis (equal). **Liping Wang:** Conceptualization (equal); supervision (equal); writing‐original draft (equal); writing‐review and editing (lead). **Yu Ke:** writing‐original draft (equal); writing‐review and editing (equal). **Hua Liao:** conceptualization (equal); supervision (equal); writing‐original draft (equal); writing‐review and editing (equal).

## Supporting information

Fig S1

Fig S2

Fig S3
