# Supplementary figures and images for: The immuno‐reactivity of polypseudorotaxane functionalized magnetic CDMNP‐PEG‐CD nanoparticles
Source: J Cell Mol Med. 2020 Nov 19;25(1):561–74. doi: 10.1111/jcmm.16109 (PMC7810964; doi:10.1111/jcmm.16109)

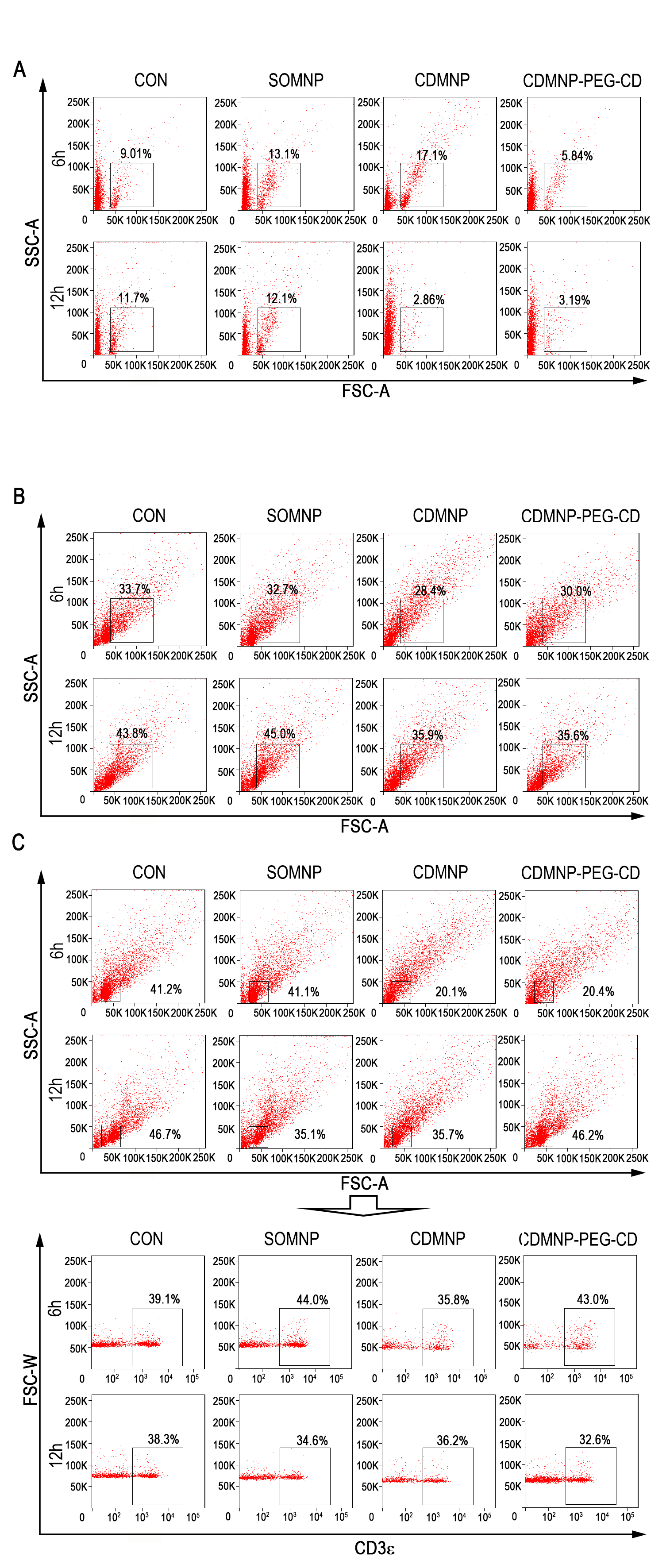

Supplement: Supplementary file 1 — Fig S1 [file JCMM-25-561-s001.tif]

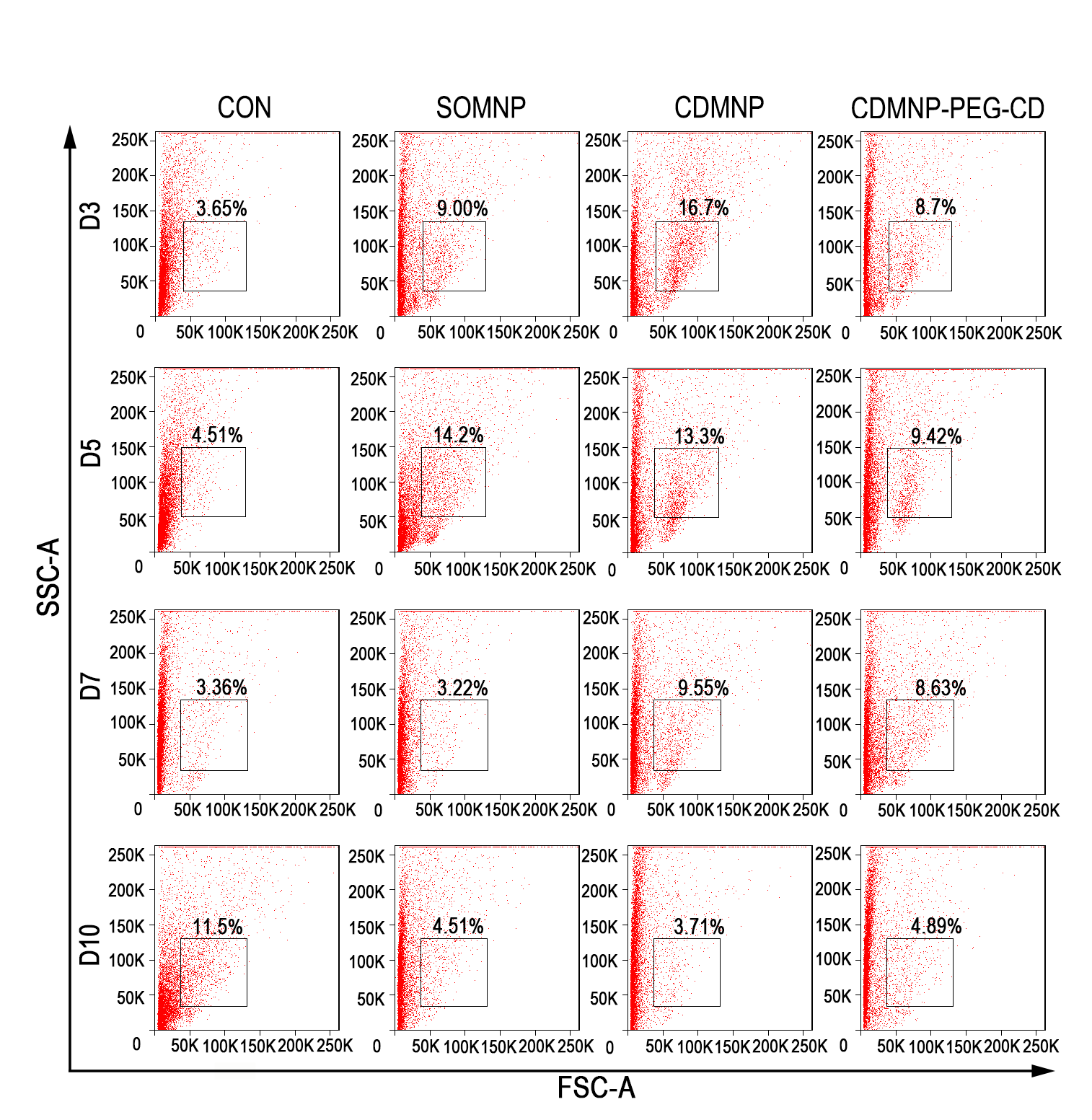

Supplement: Supplementary file 2 — Fig S2 [file JCMM-25-561-s002.tif]

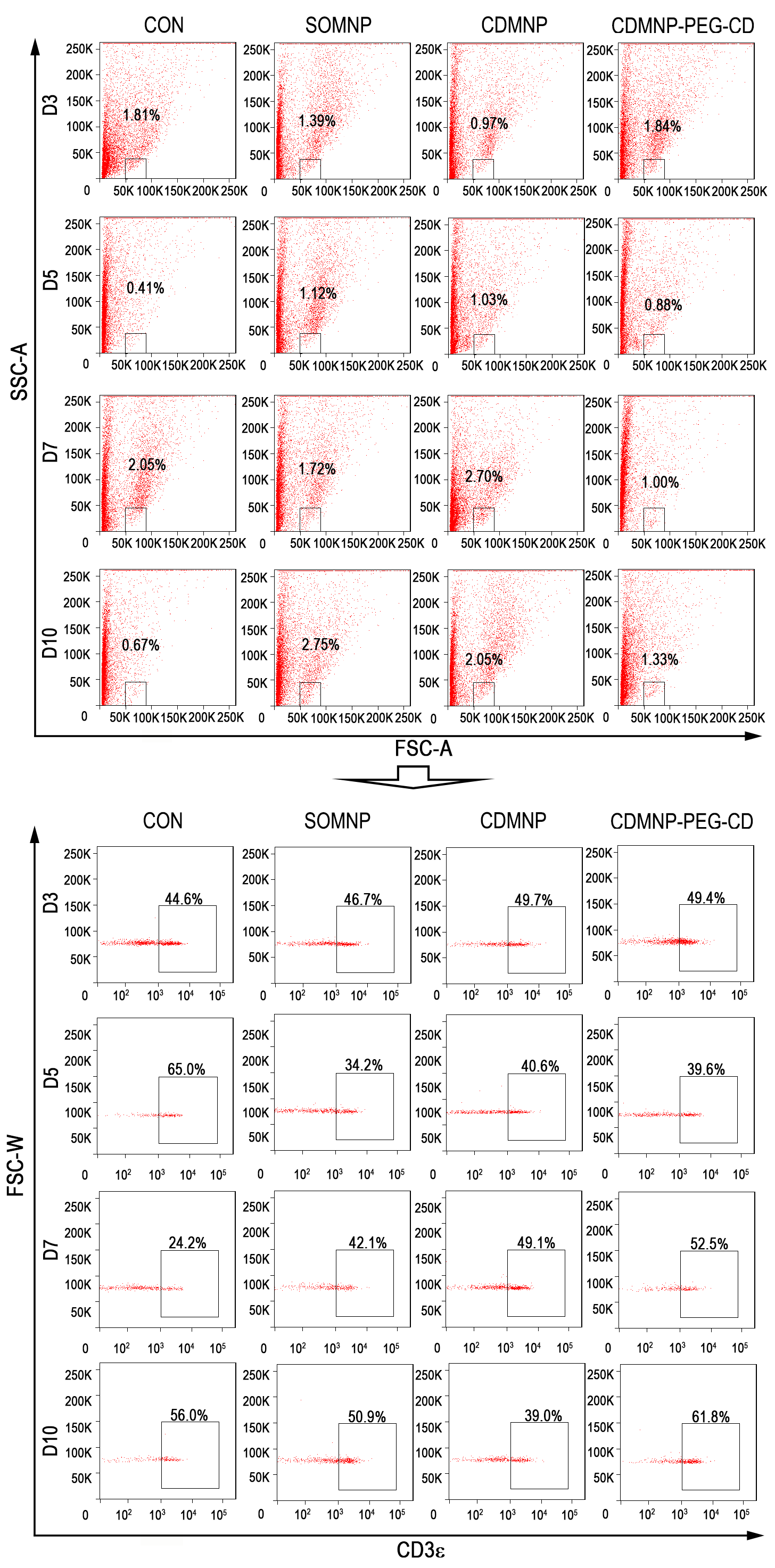

Supplement: Supplementary file 3 — Fig S3 [file JCMM-25-561-s003.tif]
